# Parkinson’s disease in real life healthcare organization database: a medication-based algorithm

**DOI:** 10.1186/s12883-026-04840-6

**Published:** 2026-03-27

**Authors:** Hila Avisar, Ruth Djaldetti, Amir Krivoy, Anat Mirelman, Roy N. Alcalay, Nir Giladi

**Affiliations:** 1 Data Research Center for Mental Health and Rehabilitation, Clalit Health Services, Petach Tikva, Israel; 2 Neurological institute, division of movement disorders, Tel-Aviv Souraski Medical center, Tel Aviv, Israel; 3 Department of Neurology, Movement Disorders Clinic, Rabin medical Center – Belinson Hospital, Petach Tikva, Israel; 4 Gray Faculty of Medical & Health Sciences, Tel Aviv University, Tel Aviv, Israel; 5 Geha Mental Health Center, Petach Tikva, Israel; 6 Department of Neurology, Columbia University Irving Medical Center, New York, NY USA

**Keywords:** Parkinson’s disease, Registry, Algorithm for identifying Parkinson’s disease, Medication- based algorithm, Parkinson’s disease incidence, Prodromal phase

## Abstract

**Background:**

Accurate identification of Parkinson’s disease (PD) in large electronic health record (EHR) population-based databases is challenging due to diagnostic heterogeneity in routine care, with a substantial proportion of individuals diagnosed with PD had not been diagnosed by a specialist. Our aim was to develop and validate a simplified rule-based medication algorithm to identify PD in a nationwide healthcare registry and apply it to estimate long-term incidence, prevalence, and pre-diagnostic diagnoses.

**Methods:**

Using Clalit Health Services EHR data covering over five million individuals (2005–2025), we constructed a medication-based algorithm incorporating predefined inclusion and exclusion criteria and two levels of diagnostic certainty (probable/possible PD). Validation was performed against two independent specialist-confirmed PD cohorts and FDOPA PET/CT and a non-PD neurological cohort. Incidence rates per 100,000 were calculated annually with 95% confidence intervals (CIs) assuming a Poisson distribution. Age-adjusted incidence rates were computed using the WHO standard population. motor and non-motor diagnoses preceding PD were examined up to 18 years before the index date using matched controls.

**Results:**

The algorithm identified 34,368 PD patients (56.5% male; mean age at index 75.2 ± 10.5 years). Sensitivity was 94.8% (95% CI 90.4–97.2) in the FDOPA PET/CT cohort, 94.8% (95% CI 92.1–96.6) in the private clinic cohort, and 94.7% (95% CI 90.9–96.9) in the movement disorder clinic cohort. Specificity was 85.2% (95% CI 77.8–90.6).

Incidence increased markedly with age but declined significantly over time (overall annual percent change [APC] - 4.47%, 95% CI -4.90 – -4.03). Age-adjusted incidence rates (≥20 years) declined 2.4-fold between 2005 and 2024 (55 [95% CI 53–58] to 23 [95% CI 21–24] per 100,000). Overall prevalence declined modestly (APC -0.78%, 95% CI -0.84 – -0.72), with increases in younger age groups and declines in older groups.

Constipation, depression, and tremor diagnoses were more frequent years before diagnosis, whereas smoking-related codes were less frequent among future PD patients.

**Conclusions:**

This validated medication-based algorithm provides a reproducible framework for PD identification in large registries. Applied over two decades in a nationwide cohort, it demonstrated high diagnostic performance and revealed age-dependent declines in PD incidence alongside heterogeneous prevalence trends.

**Supplementary Information:**

The online version contains supplementary material available at 10.1186/s12883-026-04840-6.

## Introduction

Epidemiological studies of Parkinson’s disease (PD) are often conducted in tertiary medical centers that serve a younger and more educated patient population, which may limit the generalizability of findings to the broader PD population. In contrast, real-world cohort studies based on large epidemiological databases can provide important insights into PD risk factors and epidemiology.

However, analyzing real-world data poses distinct challenges, foremost among them the accurate identification of PD diagnoses within routine clinical documentation. The challenges we faced included that a substantial proportion of individuals diagnosed with PD had not been diagnosed by a neurologist, that the level of expertise among neurologists and physicians assigning the diagnosis varied, and that diagnostic criteria changed over time. Furthermore, PD diagnoses based on real-world data may be difficult to distinguish from atypical parkinsonism (e.g., MSA and PSP) unless these diagnoses are documented in the EHR.

Criteria used to define PD differ across studies and registries [1], including reliance on diagnostic codes, medication purchases, self-reported information, visits to movement disorder clinics (MDCs), or combinations thereof [1–5].

To address these challenges, we developed a dedicated rule-based medication algorithm (hereafter referred to as the algorithm) to identify PD patients using available EHR data. This approach is based on two principles: first, that patients with PD will eventually require pharmacological treatment; and second, that computerized pharmacy purchasing databases provide a reliable means of identifying PD through the purchase of specific antiparkinsonian medications (APM) [4, 6–8].

A previous study [9], conducted in 2011 on a smaller population, proposed a medication-purchase–based algorithm. Our study follows this concept, aiming to simplify the algorithm to enhance generalizability, facilitate implementation across registries and studies, and incorporate methodological refinements to improve identification accuracy.

The algorithm was validated using independent datasets to assess its performance and generalizability. Using this approach, we analyzed individuals with index dates between 2005 and March 2025, providing a 20-year overview of incidence and prevalence trends alongside detailed demographic analyses. In addition, we examined longitudinal data on known motor and non-motor diagnoses reported to precede PD diagnosis, further supporting the validity of the algorithm and demonstrating the potential of large population-based cohorts for PD risk factor research.

The aim of this study was to develop, refine, and validate a simplified medication-based algorithm for PD identification and to apply it to characterize long-term epidemiologic trends and pre-diagnostic features in a large real- world population.

## Methods

### Participants

Israel’s mandatory National Health Insurance Law ensures that every resident is insured by one of four health maintenance organizations (HMOs). Clalit Health Services (CHS), the largest of these, insures over 50% of Israel’s population, with approximately five million active members in 2024. Since transitioning to electronic health records (EHRs) in 2000, CHS has maintained comprehensive longitudinal medical data across all care settings. The study included over five million unique individuals insured by CHS between January 2005 and March 2025, with data extracted from CHS EHRs.

The research was conducted in accordance with the Declaration of Helsinki. The study protocol was approved by the local Ethics Committee of the Geha Mental Health Center, part of CHS. Informed consent was not required due to the retrospective design and use of de-identified data.

### Data collection

Data extracted from EHRs included ICD-10 diagnoses assigned by primary care physicians, specialists, hospitals, and outpatient clinics, as well as demographics, and ATC-5 medication prescriptions and purchases.

The ethnicity analysis was classified based on their country of birth, categorized into six groups: Ashkenazi, Yemenite, Iranian-Iraqi, North African, Israeli-born, and Other (individuals born in countries not assigned to the previous categories). Socioeconomic status extracted from the database is based on the neighborhood socioeconomic score established by the Central Bureau of Statistics.

### The algorithm development process

The algorithm was developed based on a structured review of prior studies, particularly those supporting medication-based algorithms [4, 6–8], and through refinement of our previous work conducted in a smaller Israeli HMO [9]. The development process was led by a team of three movement disorder specialists with decades of clinical experience (led by N.G.), in collaboration with experts in psychiatry and data scientists from the CHS research center. In addition, we conducted a data-driven review of the cohort. This review identified specific findings that informed additional criteria. Specifically, we aimed to exclude people who received PD medications for alternative indications. For example, the identification of a large number of young women in the dataset prompted an EHR review, which confirmed dopamine agonist use for postpartum lactation suppression; this led to the formulation of criterion 4. Similarly, analysis of APM purchase patterns revealed non-continuous use, resulting in the development of criteria 13–14.

### The PD rule-based medication algorithm

The rationale for each criterion is provided in brackets in italic.

#### Inclusion criterion

- 1.  APM on at least two separate occasions (See Supplementary Table S1 for ATC-5 codes)
- 2. Had active insurance coverage with the HMO at the time of the first purchase date (FPD).
- 3. The FPD was after December 31, 2004
- 4. Age at the FPD was between 25 and 100 years.
*(The minimum of two AMP purchases is intended for individuals whose observation period is truncated by the end of the study review. Prescriptions in Israel are usually issued for a maximum of three months; hence, two purchases ensure six months of continuous use. Furthermore, patients with less than two years of follow-up are automatically classified as possible. A higher minimum requirement of APM purchases would cause us to lose more patients who are currently at the beginning of treatment. However, for individuals with longer follow-up, our algorithm requires continuous medication use, defined through several exclusion criteria (criteria 13-14) designed to ensure continuity and exclude those without sustained purchases over the years. In addition, criterion 7 excludes patients who were subsequently diagnosed with atypical parkinsonism)*

#### Exclusion criteria

Patients were excluded if they met any of the following conditions.

1. Diagnosed with hyperprolactinemia or pituitary adenoma without a PD diagnosis^1^
  *(Common clinical use of dopamine agonists for hyperprolactinemia or pituitary adenoma)*
2. Diagnosed with both pituitary adenoma and PD, and exclusively purchased bromocriptine or pergolide.
  *(Common clinical use of dopamine agonists for hyperprolactinemia or pituitary adenoma)*
3. Diagnosed with both pituitary adenoma and PD, with a FPD in the past ten years (FPD ≥2014), and exclusively purchased cabergoline.
  *(Since 2014, cabergoline use in Israel has been generally restricted to the treatment of pituitary adenoma.)*
4. Female patients who exclusively purchased dopamine agonists, had no PD diagnosis, and were either pregnant or gave birth within two years prior to the FPD.
  *(Designed to exclude female patients given dopamine agonists for postpartum lactation suppression)*
5. Diagnosed with hydrocephalus or anoxic brain injury before or up to one month after the FPD
  *(Dopaminergic medications may be used to treat hydrocephalus or anoxic brain injury, the one month prior ensures that the first APM was prescribed due to the anoxic brain injury/ hydrocephalus, and the one month after accounts for diagnoses that are logged in the system only upon hospital discharge.)*
6. Diagnosed with traumatic brain injury and coma within six months before the FPD.
  *(Dopaminergic medications may be used to treat brain injury with coma, limited to a defined time frame.)*
7. Diagnosed with progressive supranuclear palsy (PSP), multiple system atrophy (MSA), or corticobasal degeneration (CBD) without a subsequent PD diagnosis following the last such diagnosis.
  *(Excluding patients who were eventually diagnosed with atypical parkinsonism)*
8. Diagnosed with restless leg syndrome (RLS) without a subsequent PD diagnosis following the last such diagnosis.
  *(Excluding patients who were eventually diagnosed with RLS)*
9. Purchased at least one antipsychotic prescription (excluding quetiapine ≤50 mg;) during the year prior to the FPD and met either of the following:
  *(Excluding patients with drug induced parkinsonism. Quetiapine ≤50 mg is commonly prescribed for other indications and was therefore excluded.)*
  Never purchased levodopa or dopamine derivatives.
  Purchased levodopa or dopamine derivatives but were never diagnosed with PD.
    *(To avoid excluding psychiatric patients with PD)*
10. Exclusively purchased monoamine oxidase (MAO) inhibitors and met either of the following criteria:
  More than 20 purchases or a treatment duration exceeding 5 years;
  More than 8 purchases or a treatment duration exceeding 2 years; And had never been diagnosed with PD.
  *(PD progresses over time; therefore, we expect the addition of other APM medications. Accordingly, when MAO inhibitors are used exclusively for a shorter period, the case is classified as possible.)*
11. Exclusively purchased Amantadine and meet either of the following criteria:
  More than 20 purchases or a treatment duration exceeding 5 years;
  More than 8 purchases or a treatment duration exceeding 2 years; And never been diagnosed with PD
  *(PD progresses over time; therefore, we expect the addition of other APM medications. Accordingly, when amantadine is used exclusively for a shorter period, the case is classified as possible.)*
12. Never diagnosed with PD, purchased APMs for >2 years, and had a recorded diagnosis of any of the following: recurrent falls, lethargy, or general deterioration within the window of 1 year before to 5 years after the FPD.
  *(Excluding patients with long- term APM use due to alternative causes.)*
13. Never diagnosed with PD, purchased APMs for less than two years from the FPD, with more than one year having elapsed since the last APM purchase.
  *(Due to the natural history of PD progression, if there is no APM continuity and no PD diagnosis, we can conclude that the patient most likely does not have PD.)*
14. Purchased APMs for less than six months, with more than three years having elapsed since the last purchase.
  *(Due to the natural history of PD progression, if there is no APM continuity, we can conclude that the patient most likely does not have PD.)*

### Level of diagnostic certainty

PD patients were categorized into two groups: Probable PD and Possible PD.

#### Possible PD

Patients were classified as having possible PD if they met at least one of the following criteria:

- Never received a PD diagnosis.
- Fewer than two years elapsed between the FPD and the date of death, HMO exit, or data extraction
- Purchased APMs for more than three years, with more than two years since their last APM purchase.
- Diagnosed with PD and, during the year prior to the FPD, purchased at least one antipsychotic prescription and at least one prescription for levodopa or dopamine derivatives.

#### Probable PD

Patients not meeting any of the criteria for possible PD were classified as probable PD.

### Index Date

For all PD patients, the index date was defined as the earlier of the FPD of APM or the date of PD diagnosis.

Exceptions included:

- Patients diagnosed with hyperprolactinemia or pituitary adenoma prior to the FPD: For these patients, the index date was defined as the later of the FPD or PD diagnosis date.
- Patients who purchased at least one prescription for anticholinergic medications during the year prior to the FPD: For these patients, the index date was defined as the earliest of the first anticholinergic purchase date or the PD diagnosis date.
  *(As anticholinergics may precede APM as early symptomatic treatment)*

### Validation process

Patients with clinically confirmed PD were used to estimate the sensitivity (true positive rate) of the rule-based medication algorithm. Three independent external datasets were included, identified independently of the algorithm results and were not derived from the CHS database. The datasets comprised of PD diagnoses confirmed or reviewed by movement disorder specialists, each with over 20 years of clinical experience. Validation datasets were mutually exclusive; individuals appearing in more than one source were retained in a single dataset to avoid double-counting.

1. 172 PD patients who underwent FDOPA PET/CT imaging at an external medical facility, with scan results reviewed by a movement disorder specialist and restricted to clearly positive findings.
2. 225 PD patients diagnosed at the MDC at Rabin Medical Center following specialist review of medical records.
3. 383 PD patients diagnosed in a private clinic by a movement disorder specialist.

To evaluate specificity, an additional cohort of 128 patients with alternative neurological diagnoses (including gait disorders, essential tremor, essential tremor with extrapyramidal signs, enhanced physiological tremor, Ataxia, Hydrocephalus, Tourette’s syndrome, RLS, Torticollis and dyskinesia), diagnosed by a movement disorder specialist, was analyzed. This cohort was confirmed to be non-overlapping with the PD validation datasets. Specificity and false positive rate were calculated within this cohort. Confidence intervals (CIs) at the 95% level were calculated using the Wilson method.

Clinical diagnoses established by experienced movement disorder specialists were used as the reference standard. Neuropathological confirmation represents the definitive gold standard for PD diagnosis [10] ; however, such data were not available in this population-based study.

Identifiable patient lists were transferred to the CHS computer department for secure linkage. Only de-identified system identifiers were returned to the research team, and all analyses were conducted exclusively using de-identified data.

An alternative diagnosis-based approach was examined to evaluate the completeness of PD case ascertainment based solely on diagnostic coding and to assess specialist involvement. All individuals who received a first PD diagnosis between 2005 and 2024 were included. We calculated the proportion of patients with at least one PD diagnosis assigned by a neurologist and identified visits to a MDC, which may indicate specialist evaluation even if the PD diagnosis was not explicitly documented by a neurologist in the EHR. To examine temporal trends, we analyzed, for each calendar year for our PD cohort, the proportion of patients diagnosed by a neurologist within the first year after the index date.

We further assessed the proportion of patients who purchased at least one APM through December 2025. Analyses were performed for the entire diagnosis-based cohort and separately for two subgroups: (1) patients diagnosed by a neurologist or who visited an MDC, and (2) patients without a documented neurologist diagnosis and no MDC visit.

### Motor and non-motor diagnoses in the years before index date

To examine the pre-diagnostic phase of PD and to provide additional support for the validity of the cohort, we analyzed selected motor and non-motor diagnoses known to precede PD: Constipation (K59), Tremor (G25.0), Tremor, not other specified (NOS) (R52.1), Depressive episode (F32), Mental and behavioral disorders due to use of tobacco (F17) Heavy smoker (Z72.0). For each year relative to the index date, ranging from the year immediately prior to diagnosis (noted as year 0) up to 18 years before the index date (noted as year − 17). The proportion of subjects receiving at least one diagnosis within every year was calculated relative to the total number of individuals with available data for that year. To allow comparison between PD patients and controls, accounting for age the artificial Index date of the controls were determined by their matched PD patient index date [2].

Analyses were conducted overall and stratified by age at index (age at index ≤ 75 / age at index > 75) and by sex. Differences in the prevalence of each diagnosis between cases and controls were assessed using chi-squared tests for independence. To account for multiple comparisons, false discovery rate (FDR) correction was applied [11].

Controls were defined as CHS members who had no record of purchasing APMs and were never diagnosed with PD. Controls were matched to cases at a 1:4 ratio without replacement, using exact matching for sex, age (± 1 year), and sector and ethnic group were matched using propensity scores to ensure balance. To address potential bias due to censoring, controls were required to be alive at the index date of their matched PD case. Additionally, controls were matched on data availability, ensuring equivalent follow-up duration to that of the corresponding PD patient. The same (artificial) index date as the matched PD case was assigned to each control, allowing outcomes and test results to be analyzed at equivalent ages for cases and controls.

### Incidence and prevalence

Annual crude incidence rates per 100,000 population were calculated for the following age groups: [20–40), [40–50), [50–60), [60–70), [70–80), [80–90), and [90–100), by dividing the number of new PD cases recorded in each calendar year by the corresponding age-specific insured population in that year. Given the comprehensive and stable population coverage of CHS, annual population counts were considered a close approximation of person-time at risk.

Age-adjusted incidence rates (AAIRs) were computed using the direct standardization method, based on 5-year age groups of the World Health Organization (WHO) standard population [12]. We additionally calculated AAIRs for ages ≥ 20 using the same standard population truncated at age 20 and renormalized to 100%, as this represents the population at risk. For both crude and age-adjusted incidence rates, 95% CIs for incidence rates were calculated assuming a Poisson distribution of the observed case counts. For the temporal trends, overdispersion was assessed using the deviance-to-degrees-of-freedom ratio, and models were fitted using a quasi-Poisson approach when overdispersion was detected.

Annual point prevalence for the years 2000–2024 was calculated by dividing the number of algorithm-defined PD cases in each calendar year by the corresponding annual insured population. Estimates were generated for the total population and stratified by sex. Temporal trends in prevalence were assessed using binomial regression with calendar year modeled as a continuous variable and weighted by annual population size. Models were fitted overall and stratified by age group. Annual percent change (APC) with 95% CIs was derived from the regression coefficients for both incidence and prevalence trend models.

## Results

**Fig. 1:**
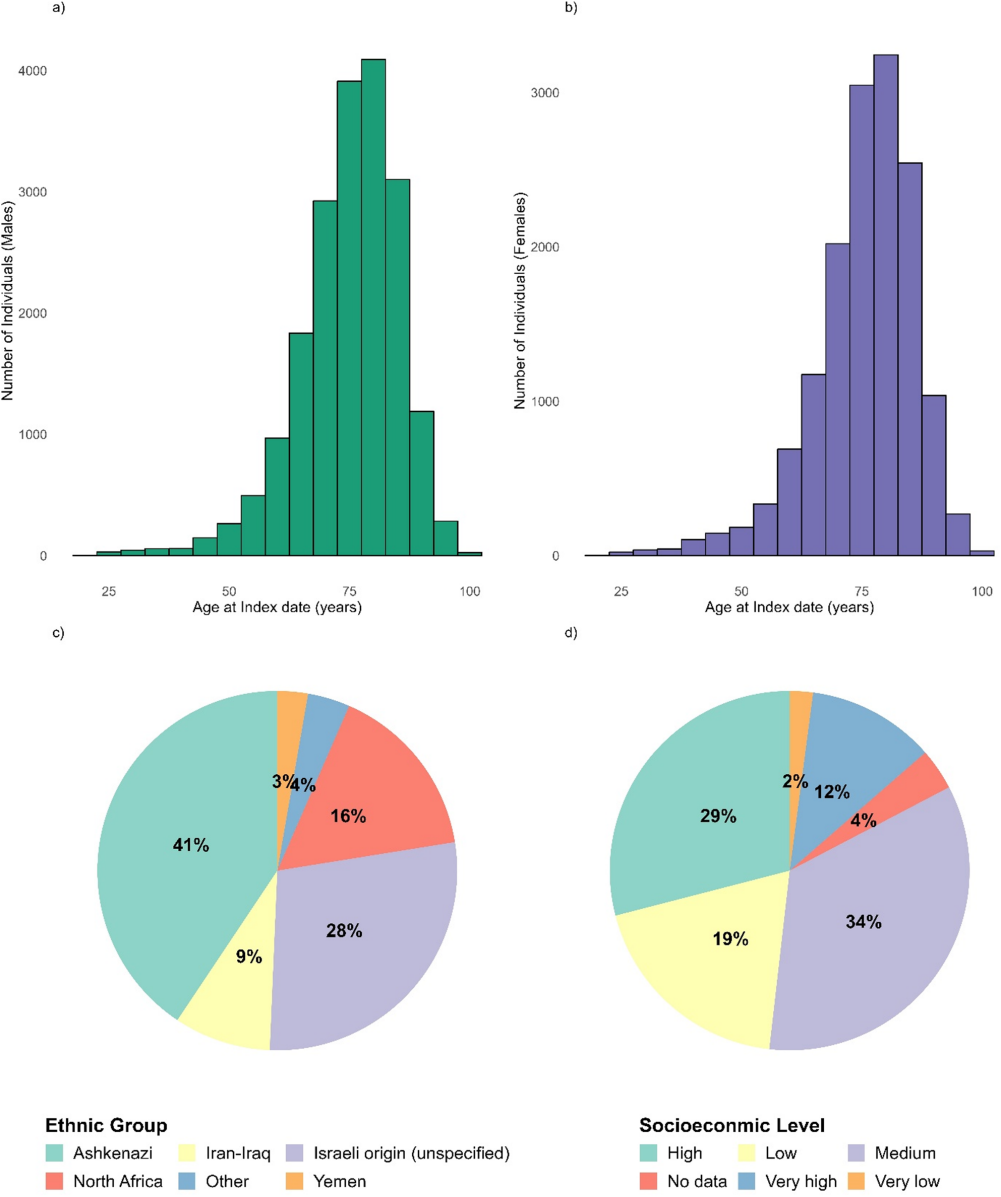
**(A)** Age at index distribution for Males (**B**) Age at index distribution for females (**C**) Ethnicity distribution by their country of birth. Individuals born in Israel cannot be categorized. (**D)** Socioeconomic distribution

A total of 34,368 individuals from approximately 5 million people between 2005 and 2024 met all inclusion criteria and were classified as patients with PD, with an index date on or after January 1, 2005. Of these, 56.5% were male and 43.5% female, and 13,090 patients were alive at the time of analysis (March, 2025). The average age at index was 75.2 years (SD = 10.5) overall, and 70.0 years (SD = 11.5) among those alive. Age of index distribution of males and females was similar (Fig. 1A-B). Table 1 presents the final cohort characteristics, stratified by sex and PD diagnostic certainty (probable and possible). Supplementary Table S2 outlines the algorithmic selection process based on the inclusion and exclusion criteria, Criterion 4 (female patients who exclusively purchased dopamine agonists without a PD diagnosis and with pregnancy or childbirth within the two years preceding the FPD) contributed the largest proportion of exclusions, representing approximately 50% of the initial female cohort. The cohort is ethnically diverse, with 72% born outside of Israel (Fig. 1C). 6.8% are Arabs (94% were born in Israel). The cohort is also characterized with relatively high socioeconomic status, with 41% defined as high and very high compared to 21% with low and very low socio socioeconomic status (Fig. 1C).

**Table 1:** Characteristics of the PD patients

Characteristics	Males		Females		All	
	Total	Alive	Total	Alive	Total	Alive
Probable:						
Number	12,839	4878	9091	3796	21,930	8674
Age at Index date (years), mean (SD)	74.3 (9.6)	70.0 (10.1)	75 (9.6)	70.5 (10.2)	74.5(9.6)	70.2 (10.1)
Possible:						
Number	6596	2275	5842	2141	12,438	4416
Age at Index date (years), mean (SD)	76.2 (11.6)	70.1 (13.5)	76.3 (12.1)	69.4 (13.9)	76.2 )11.8)	69.8 (13.7)
Total:						
Number	19,435	7153	14,933	5937	34,368	13,090
Age at Index date (years), mean (SD)	75 (10.3)	70.0 (11.3)	75.5 (10.7)	70.1 (11.7)	75.2 (10.5)	70.0 (11.5)

We defined two diagnostic confidence levels: Probable (high probability) and Possible.

Overall, 64% of PD patients were classified as probable, with similar proportions by sex: 66% of males and 61% of females.

To be classified as possible, patients were required to meet at least one of the predefined criteria (Supplementary Table S3). Among those categorized as possible, 29% met two or more criteria, and 55% had less than two years of follow-up. While 31% had no recorded PD diagnosis, only 12% were defined possible based solely on this criterion. Notably, 48% of those without a PD diagnosis also had less than two years of follow-up.

### Validation results

Across three independent PD-confirmed cohorts, the algorithm demonstrated consistently high sensitivity. For sensitivity estimation, cases classified as Probable PD and Possible PD were combined and considered PD. Sensitivity was ~ 95% across all datasets (Table 2). Most PD cases not identified by the algorithm had purchased less than one APM. The remaining non-identified cases were excluded according to the algorithm criteria (Supplementary Table S4).

**Table 2:** Diagnostic performance of the rule-based medication algorithm across independent validation cohorts

Dataset	*N*	Sensitivity (%)	95% CI
FDOPA PET/CT	172	94.8%	[90.4, 97.2]
Private clinic	383	94.8%	[92.1, 96.6]
MDC	225	94.7%	[90.9, 96.9]

Dataset	N	Specificity (%)	95% CI
Private clinic - Mimic cohort	128	85.2%	[77.8, 90.6]

To assess specificity, 128 patients with alternative neurological diagnoses were evaluated. Nineteen individuals were misclassified as PD, yielding a specificity of 85.2% (95% CI 77.8–90.6) and a false positive rate of 14.8% (95% CI 9.4–22.2).

Detailed classification outcomes and distribution across algorithm exclusion criteria are presented in Supplementary Table S4. All validation datasets were mutually exclusive.

Analysis of the alternative diagnosis-based approach demonstrates that among the 65,856 individuals diagnosed with PD between 2005 and 2024, 56.3% had no documented PD diagnosis assigned by a neurologist. After excluding individuals who had visited a MDC, including visits prior to their first PD diagnosis, 49.9% had neither a neurologist diagnosis nor a MDC visit. These figures do not account for private neurologists’ visits, which are not documented in the EHR. Over time, however, specialist involvement increased, as the proportion of patients diagnosed by a neurologist within the first year after the index date rose 1.53-fold between 2005 and 2023 (Supplementary Fig. S1).

Overall, 44.2% of patients who received a PD diagnosis did not purchase any APM through the end of 2025. This proportion was substantially higher among patients with neither a neurologist diagnosis nor a MDC visit (52.6%) compared to those with documented specialist involvement (19.8%).

### Motor and non-motor diagnoses in the years before index date

**Fig. 2:**
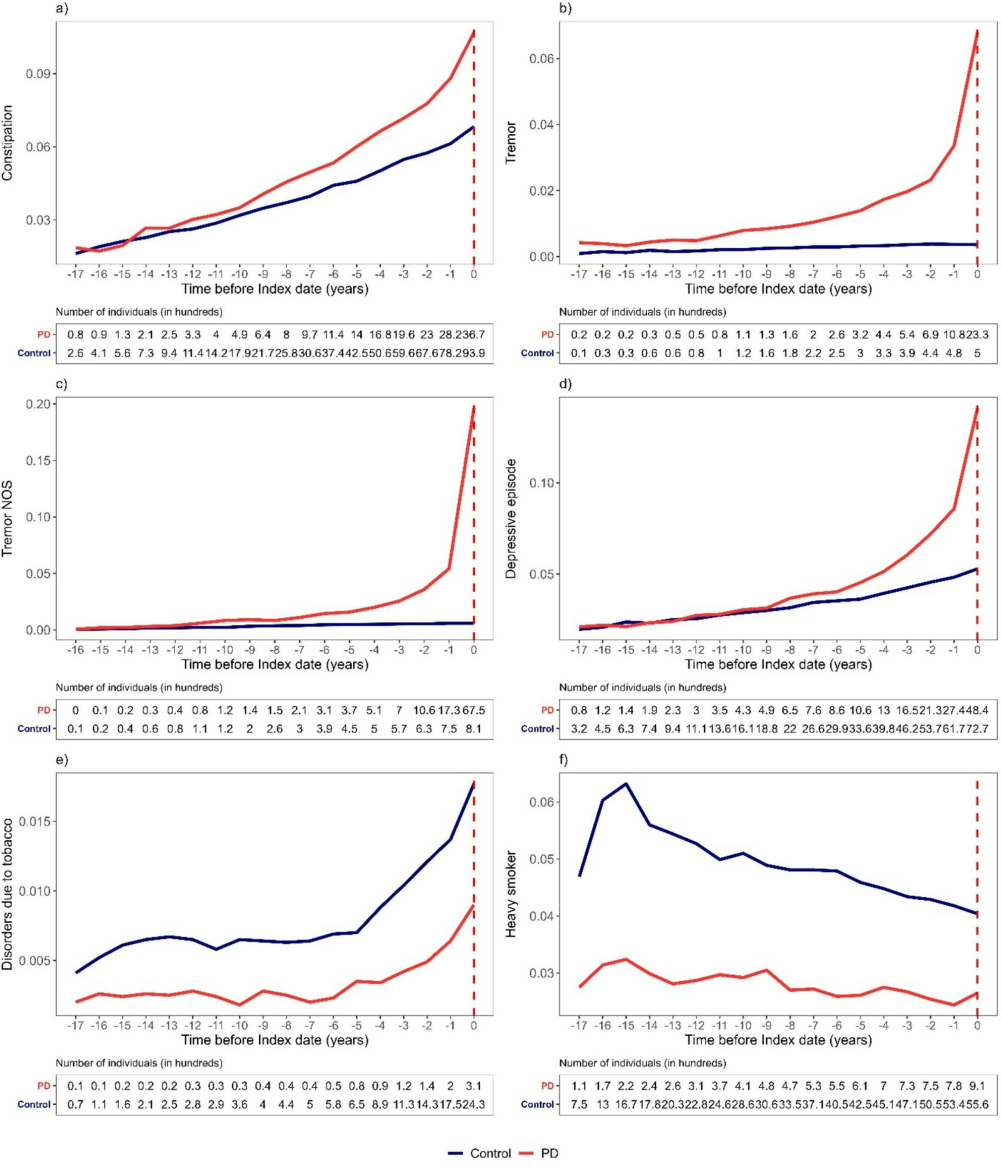
Annual diagnosis prevalence for PD patients and controls in the years before the Index date (red dashed vertical line). The results are calculated based on data collected in the previous year; for example, “-5” relates to the mean blood test value calculated during the sixth year before index date. For controls, the Index date is defined by the index date of their matched patient with PD. (**A)** Constipation (K59). (**B)** Tremor (G25.0). (**C)** Tremor NOS (R52.1). (**D)** Depressive episode (F32). (**E)** Mental and behavioral disorders due to use of tobacco (F17). (**F) **Heavy smoker (Z72.0)

Figure 2 demonstrates the prevalence of known motor and non-motor diagnoses associated with PD among PD patients compared with controls in the years before the index date. Supplementary Figure S2 highlights the time periods during which these differences are statistically significant, stratified by sex and by age at index date (before vs. after age 75). Supplementary Table S5 provides the p-values, FDR-adjusted p-values, and CIs for each year.

**Fig. 3:**
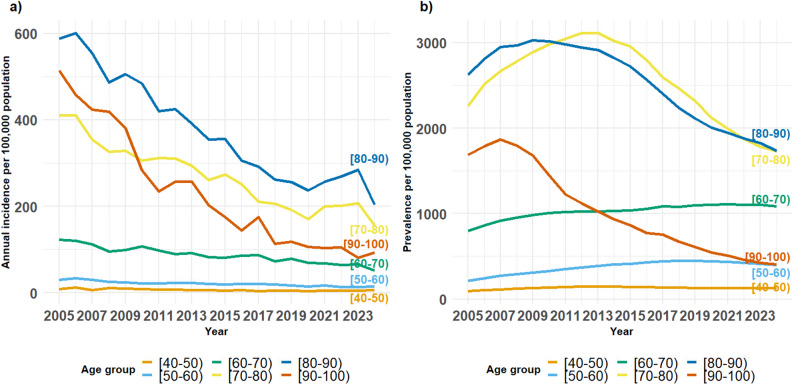
(**A**) Annual incidence rate of PD per 100,000 CHS insured individuals from 2005 to 2024, stratified by age group (**B**) Annual prevalence for PD for CHS insured individuals from 2005 to 2024

Constipation was associated with PD (Fig. 2A). PD patients had a significantly higher prevalence of constipation starting 10 years before the index date (12 years in males), reaching a 4% difference in the final year before diagnosis (FDR-adjusted *p* < 0.001; 95% CI 0.035–0.042). The prevalence of a depressive episode (Fig. 2B) differed significantly between PD patients and controls starting 9 years before the index date, reaching its peak in the final year with a 9% difference (FDR-adjusted *p* < 0.001; 95% CI 0.08 − 0.09), with a more pronounced difference among patients with an index date after age 75. PD patients were more likely to receive a tremor diagnosis years before the index date. Tremor NOS (Fig. 2C) and tremor (Fig. 2D) diagnoses were more common among those later diagnosed with PD as early as 16 and 18 years before diagnosis, in men and women respectively (Supplementary Fig. S2), with differences ranging from 1% to 5% and peaking in the final year before diagnosis. Regarding smoking-related codes, mental and behavioral disorders due to tobacco use (Fig. 3E) and heavy smoking (Fig. 3F) were, as expected, less prevalent among PD patients than controls, extending up to 18 years before the index date in males. For example, there was 3% difference 16 years before diagnosis (FDR-adjusted *p* < 0.001; 95% CI -0.04 – -0.03).

#### Incidence and prevalence

Table 3; Fig. 3A present the annual incidence rates (new PD cases per 100,000 insured individuals) stratified by age group for the years 2005–2024. Incidence rates declined significantly over the study period. In the overall population, the APC was − 4.47% (95% CI: -4.90% – -4.03%; *p <* 0.001). Age-stratified analyses demonstrated significant declines across all age groups: -3.42% (95% CI: -6.11% –-0.65%) in ages [20–40), -4.27% (95% CI: -5.66% –-2.85%) in [40–50), -4.12% (95% CI: -4.66% –-3.57%) in [50–60), -3.52% (95% CI: -4.00% –-3.04%) in [60–70), -4.50% (95% CI: -5.05% –-3.94%) in [70–80), -5.33% (95% CI: − 5.91% –-4.75%) in [80–90), and − 9.60% (95% CI: -10.34% –-8.86%) in [90–100) (all *p* < 0.001). The largest relative decrease was observed among individuals aged [90–100). Age-specific trends were similar when stratified by sex (Supplementary Fig. S3). This pattern was more pronounced in older age groups and is also reflected in the difference between the cohort’s average age at index date (75.2), compared to the average age for those alive (70.0).

**Table 3:** Crude annual PD incidence rate stratified by age group and AAIR for ages 20–100 for the years 2005–2024

Age group/ Year	Crude PD incidence rate per 100,000 individuals (CI) *							AAIR	
	[20–40)	(40–50)	[50–60)	[60–70)	[70–80)	[80–90)	[90–100)	AAIR	AAIR (≥ 20)
2005	2 (1,2)	9 (6,12)	30 (25,35)	122 (109,135)	410 (384,436)	588 (545,630)	514 (426,601)	36 (35,38)	55 (53,58)
2006	2 (1,3)	12 (9,15)	33 (28,39)	120 (107,133)	410 (384,436)	600 (558,642)	458 (375,542)	37 (36,39)	57 (55,59)
2007	2 (1,3)	7 (4,9)	32 (25,36)	112 (100,125)	355 (331,379)	554 (514,594)	424 (341,506)	33 (32,35)	51 (48,53)
2008	1 (1,2)	11 (7,14)	26 (21,31)	95 (84,106)	327 (303,350)	487 (450,524)	419 (337,501)	30 (29,31)	46 (44,48)
2009	2 (1,2)	10 (7,13)	24 (19,29)	99 (88,111)	329 (306,352)	505 (468,543)	381 (304,458)	31(29,32)	47 (45,49)
2010	1 (1,2)	9 (6,12)	22 (18,26)	108 (97,119)	306 (283,329)	484 (448,520)	284 (220,348)	29 (28,31)	45 (43,47)
2011	1 (0,1)	7 (4,10)	22 (18,27)	98 (88,109)	312 (289,335)	420 (386,453)	235 (178,291)	28 (26,29)	42 (40,44)
2012	1 (1,2)	8 (5,10)	24 (19,28)	90 (80,99)	311 (311, 334)	425 (391,458)	258 (200,315)	27 (26,28)	41 (39,43)
2013	1 (1,2)	7 (4,9)	23 (18,28)	92 (83,102)	294 (272,317)	392 (360,424)	257 (202,312)	26 (25,27)	40 (38,42)
2014	1 (1,2)	6 (4,8)	21 (17,26)	83 (74,92)	261 (240,282)	355 (324,386)	202 (155,249)	23 (22,25)	36 (34,38)
2015	1 (0,1)	5 (3,7)	19 (15,24)	82 (73,91)	274 (253,295)	356 (326,387)	175 (132,218)	23 (22, 25)	36 (34,38)
2016	1 (0,1)	7 (4,9)	20 (16,25)	86 (77,95)	251 (231,271)	306 (277,334)	145 (107,183)	22 (21,23)	34 (32,36)
2017	1 (0,1)	4 (2,5)	20 (16,25)	87 (78,96)	211 (193,229)	292 (265,319)	176 (134,217)	20 (19, 22)	31 (30,33)
2018	1 (0,1)	5 (3,7)	20 (15,24)	72 (64,81)	206 (189,223)	262 (236,288)	113 (80,146)	19 (18,20)	29 (27,31)
2019	1 (0,2)	5 (3,7)	18 (14,22)	79 (70,87)	192 (176,208)	255 (230,281)	119 (85,152)	19 (18,20)	29 (27,30)
2020	1 (0,1)	5 (3,6)	15 (11,19)	69 (61,78)	170 (155,185)	237 (213,262)	106 (75,137)	17 (16,18)	25 (24,27)
2021	1 (0,1)	5 (3,7)	17 (13,21)	68 (60,77)	200 (184,216)	257 (232,283)	104 (73,135)	18 (17,19)	28 (26,29)
2022	1 (0,1)	5 (3,6)	14 (10,17)	65 (57,73)	201 (186,217)	269 (243,295)	105 (74,136)	18 (17,19)	27 (26,29)
2023	1 (1,2)	5 (3,6)	14 (10,18)	66 (58,74)	207 (191,222)	284 (257,311)	82 (54,109)	18 (17,19)	28 (26,29)
2024	2 (1,3)	6 (4,8)	15 (11,18)	52 (45,59)	156 (143,169)	204 (181,227)	93 (63,122)	15 (14,16)	23 (21,24)

(*) Crude annual PD incidence rate with 95% CI stratified by age group for the years 2005–2024 and Age adjusted incidence rate (AAIR) for ages 20–100, based on World Health Organization

The AAIRs were calculated for each year for the total population (ages 0–99) range between 15 and 36, with a 2.4 -fold reduction between 2005 and 2024 and for the population over the age of 20 (the population at risk) ranges between 23 and 55 with a 2.4-fold reduction (The decrease observed in 2020 may be attributed to the COVID-19 pandemic, during which individuals sought medical care primarily for urgent matters.).

Table 4 and shows the number of PD patients and the prevalence for every age group stratified by sex. Analyzing the prevalence trends, show that overall prevalence declined significantly over the study period, with an annual percent change (APC) of -0.78% (95% CI -0.84% – -0.72%, *p <* 0.001). Figure 3B; Table 4 Age-stratified analyses revealed heterogeneous trends. Prevalence increased among younger age groups (e.g.,50–60 years: APC 2.93%, 95% CI 2.73% – 3.13%), while significant declines were observed in older age groups (e.g., 70–80 years: APC − 2.23%), A similar pattern was observed in both sexes (Supplementary Fig. S4).

**Table 4:**
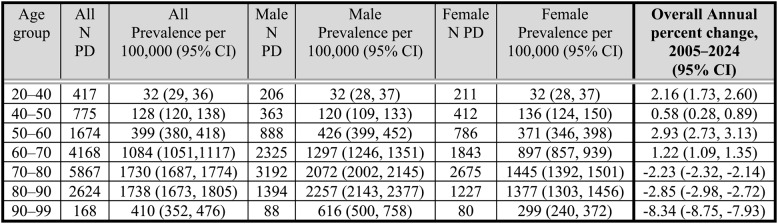
Age and sex-specific prevalence of PD per 100,000 population in 2024 and annual percent change (2005–2024)

## Discussion

Using a novel medication-based approach to PD identification, based on previous studies, incorporating data driven analysis and consultation with a team of movement disorder specialists, we developed an algorithm suitable for large-scale cohorts based on EHRs data. The algorithm incorporates a clearly defined index date and two levels of diagnostic certainty. Applying this approach to a real-world cohort of over five million individuals, we estimated the incidence and prevalence of PD.

Validation against clinically defined sub-cohorts, including patients evaluated by movement disorder specialists and those who underwent a FDOPA PET/CT imaging, demonstrated high sensitivity of the algorithm. Our findings are consistent with prior literature, showing increasing prevalence with age, higher prevalence in men [5, 8, 13–16], and the expected pattern of motor and non-motor diagnoses in the years preceding PD diagnosis compared with controls.

Most antiparkinsonian medications are unique to PD, and for those also prescribed for other conditions such as dopamine agonists, amantadine, and MAO inhibitors, our algorithm applies clearly defined exclusion criteria to distinguish between PD and other diseases. Pharmacy purchase records, particularly in populations with universal drug coverage, provide a robust and precise method for tracking medication use. Maintained for administrative purposes, these databases are regularly validated, thereby supporting large-scale, population-based observational studies with extended follow-up, reduce selection bias, and enhance generalizability.

In our approach, medication use was confirmed by requiring at least two separate purchase events, ensuring that inclusion was not based on a single prescription. While smaller cohort studies can confirm diagnoses through multiple clinical pathways, population-wide studies require EHR-based algorithms with high diagnostic accuracy and minimal bias. Restricting case identification solely to neurologists’ diagnoses is problematic: our analysis demonstrated that 50% of people with recorded PD diagnosis in their EHRs, had neither a neurologist-recorded diagnosis nor an MDC visit. We also risk excluding individuals treated in private clinics, where diagnoses may be documented by primary care physicians, or may not be captured in the EHR at all. However, PD patients treated in private clinics will almost always purchase through the HMO, since all Israeli residents are covered by Israel’s national health insurance. Furthermore, the finding that 44% of patients who received a PD diagnosis did not purchase APM, a non-concordance with PD natural history, highlights the shortcomings of a diagnosis-based algorithm.

This approach is consistent with several other studies in diverse settings [4–6, 8, 15, 17] that have incorporated APM use into PD definitions. Wong et al. [5], for instance, classified PD based on one diagnostic code for parkinsonism plus one drug claim; Our algorithm addresses such cases differently, applying additional exclusion criteria and classifying individuals without a diagnostic code as “possible” PD. Similarly, a nationwide Norwegian study [15] defined PD solely through dopaminergic drug prescriptions. A recent study based on the California PD Registry [18], the largest state-wide PD registry, requiring mandatory electronic reporting of all eligible cases, identified four major gaps in population-based PD registries. One of the most critical gaps was case definition, with estimates suggesting that only 75–82% of cases detectable by diagnostic codes actually represent PD [19–21] .

The classification of PD diagnoses into “Probable” and “Possible” categories enables stratification of the cohort according to the level of diagnostic certainty. This distinction provides analytical flexibility, allowing analyses to focus on a subgroup with higher diagnostic certainty or to include the broader cohort to increase statistical power, while acknowledging potential implications for specificity and generalization.

The mean age at index was 75.2 years overall, compared to 70 years among individuals still alive at the time of analysis. The mean age at diagnosis is higher than that reported in smaller studies but aligns with recent population-based studies [5, 14, 15]. For example, a Canadian study based on ~ 10.5 million individuals reported a mean diagnostic age of 72.6 years population [5] .

The analysis of motor and non-motor diagnoses in the pre-diagnosis phase of PD had two complementary aims: to further validate the cohort and case-identification approach, given the expectation of differential prevalence of these diagnoses among PD patients compared with controls, and to use the long follow-up and large sample size to delineate the temporal pattern of divergence between the two groups across the years preceding diagnosis. We therefore compared the prevalence of these diagnoses between PD patients and controls examining trends up to 20 years prior to the index date. Consistent with previous studies, constipation [22, 23] and depressive episode [24–26] were significantly more prevalent in the PD group up to a decade before the index date. In addition, tremor diagnoses (including Tremor NOS) [27] were more common as early as 18 years prior, alongside an inverse association for mental and behavioral disorders related to tobacco use and heavy smoking [28–31]. Collectively, these findings support the validity of the cohort and methodology, and demonstrate how population-based, stratified longitudinal analyses can provide further insight into the evolution of clinical features in the years leading up to PD diagnosis. These analyses are descriptive and report diagnoses recorded in the EHR prior to the index date; they are conditional on healthcare documentation and clinical practice and are not intended to distinguish biological prodromal manifestations from healthcare-related signals.

A limitation of the medication-based approach is the potential under-ascertainment of patients in the earliest stages of disease who have not yet initiated pharmacological treatment. Although most patients begin treatment within months of diagnosis, some early or atypical cases may not be captured, potentially leading to conservative incidence estimates. Other reasons for underdiagnosis would be (1) Individuals with PD, who were never correctly diagnosed clinically and therefore never treated; and (2) PD patients that purchased the APM outside the HMO pharmacy system, which is unlikely given the excessive long-term cost of purchasing these medications outside the HMO system. Furthermore, to mitigate misclassification, and minimize over diagnosis we excluded alternative indications for antiparkinsonian medications (e.g., drug-induced parkinsonism, lactation suppression), and competing neurological diagnoses, such as MSA and PSP. The algorithm also required sustained or repeated treatment, particularly in individuals without a recorded PD diagnosis. While these criteria enhance specificity, they may inadvertently exclude a small number of true PD cases with overlapping conditions.

Case identification was performed algorithmically using administrative data, minimizing observer bias. Incorporation bias is minimized, as validation datasets were based on independent specialist clinical diagnoses and on an FDOPA PET/CT imaging dataset rather than prescription data. However, because validation relied primarily on specialist-managed and imaging-confirmed cases, spectrum and verification biases may be present, potentially leading to overestimation of diagnostic sensitivity and limiting generalizability of the performance estimates. Another limitation is that neuropathological confirmation, which represents the definitive gold standard for PD diagnosis [10], is not available in Israel. Therefore, clinical diagnoses used for validation were established by experienced movement disorder specialists or by FDOPA PET/CT imaging results reviewed by a specialist. In the future, if tissue biomarkers become more widely available, we advise re-validating the algorithm.

In addition, healthcare utilization and diagnostic timing may vary over time, which can influence both PD diagnosis coding and initiation of antiparkinsonian treatment in administrative data. For example, reduced outpatient evaluations during the COVID-19 pandemic may have delayed diagnostic workup and treatment initiation. The increasing proportion of individuals diagnosed by a neurologist within the first year following the index date over the years, as demonstrated in our study, may contribute to improved PD identification and treatment. CHS, the largest HMO, serves a relatively older population, particularly in the age group above 65 years. Given the strong age-dependence of PD incidence and prevalence, age-stratified analyses are therefore essential when interpreting trends. Finally, another important factor to consider is that in Israel, under the universal coverage framework, all medically necessary services are included in the national health basket; neurologist consultations, MDC visits, and APM are publicly covered. While some individuals also hold supplementary or private insurance that enables consultation with private specialists, medication dispensing remains primarily conducted through the HMOs, as pharmacological treatment is fully covered within the public system. This structure might vary from other countries in terms of access to physicians for diagnostic and treatment, and may decrease the impact of other factors such as socioeconomic status. Furthermore, this structure allows comprehensive capture of medication-based case identification within administrative HMO data, even when part of the diagnostic process occurs in the private sector. Hence, because medication dispensing is comprehensively captured within Israel’s universal coverage framework, the performance and generalizability of medication-based case ascertainment may differ in healthcare systems outside Israel with fragmented pharmacy benefits or substantial out-of-pocket medication purchasing; therefore, local validation is recommended before implementation in other settings.

Our Incidence findings concur with prior studies showing that PD incidence increases with age [1, 15, 32–35]. Analysis of incidence rates over the last two decades demonstrated a declining trend, most pronounced in the older population. Reports on temporal incidence trends remain inconsistent. Several large population-based studies from Taiwan, Canada, the United Kingdom, Germany, Korea, and the Netherlands have described declining incidence, particularly in older age groups populations [5, 8, 36–39], whereas others have reported increasing incidence globally [40] or within specific countries [8, 35, 41–45].

The decline in incidence observed in our older population may reflect, at least in part, improved diagnostic precision over time. This interpretation is supported by our finding that the proportion of individuals diagnosed by a neurologist within the first year following the index date increased 1.53-fold between 2005 and 2023, suggesting greater specialist involvement and potentially reduced misclassification of parkinsonian syndromes. Alternatively, the observed decline in incidence may reflect a true decline in PD incidence in Israel, which may be explained by improvements in health behaviors (e.g., physical activity), reduced exposure to environmental risk factors such as pesticides [46], or other factors that should be further studied. Future research should investigate the reasons underlying the observed decline in incidence over time.

Although incidence declined significantly across age groups, prevalence trends were heterogeneous. Because prevalence reflects both incidence and disease duration, declining incidence does not necessarily translate into parallel reductions in prevalence. The increase observed in younger age groups may reflect improved survival or earlier diagnosis, resulting in longer disease duration, whereas the marked decline in older age groups may reflect both reduced incidence and competing mortality. Together, these findings suggest that temporal changes in PD burden in this population involve shifts in both case ascertainment and age-specific survival dynamics.

In conclusion, we present a validated, medication-based algorithm for identifying PD patients, specifically designed for use in large-scale registries to enhance accuracy and address potential biases. Implemented and validated in a nationwide population over a 20-year period, the algorithm demonstrated its robustness and yielded detailed insights into PD incidence, prevalence, patient characteristics stratified by age and sex. It also enabled longitudinal analyses of motor and non-motor diagnoses preceding PD diagnosis.

This large, validated cohort enables testing the effects of environmental and pharmacological interventions on PD risk leveraging over two decades of EHR data, for example, the use of calcium channel blockers or GLP-1 analogs. Moreover, it provides a solid foundation for utilizing artificial intelligence (AI) methodologies to incorporate potential risk factors derived from the EHRs (e.g., blood test results, medication purchases, and other diagnoses) in order to identify individuals at elevated risk for PD up to 20 years before diagnosis.

## Supplementary Information

Supplementary Material 1

## Data Availability

Data was extracted from Clalit Health Services (CHS) using Clalit’s data-sharing platform powered by MDClone, ensuring anonymity. Permission to use this dataset for the current analysis was granted by CHS HMO. The dataset for our study, along with the data dictionary, and full documentation, are securely held by CHS HMO. Due to ethical restrictions, data for the current study cannot be shared.The underlying code for this study is not publicly available but may be made available to qualified researchers on reasonable request from the corresponding author.

## Footnotes

1 PD diagnosis include: PD, Dementia in PD, Extrapyramidal disease, Movement disorders, Parkinsonism idiopathic, Parkinsonism, Secondary Parkinsonism.

## Abbreviations

Abbreviation: Definition
AAIR: Age-adjusted incidence rate
AI: Artificial intelligence
APC: Annual percent change
APM: Anti- parkinsonian medication
CBD: Corticobasal degeneration
CHS: Clalit Health Services
CI: Confidence interval
FDR: False discovery rate
FPD: First purchase date
EHR: Electronic health records
HMO: Health maintenance organizations
MAO: Monoamine oxidase
MDC: Movement disorder clinic
MSA: Multiple system atrophy
NOS: Not otherwise specified
PD: Parkinson’s disease
PSP: Progressive supranuclear palsy
RLS: Restless leg syndrome
SD: Standard deviation
TPR: True positive rate
WHO: World Health Organization
