# Supplementary material for: Parkinson’s disease in real life healthcare organization database: a medication-based algorithm
Source: BMC Neurol. 2026 Mar 27;26:327. doi: 10.1186/s12883-026-04840-6 (PMC13185355; doi:10.1186/s12883-026-04840-6)
Supplement: Supplementary file 1 — Supplementary Material 1 [file 12883_2026_4840_MOESM1_ESM.pdf]

Parkinson’s disease in real life healthcare organization database: a medication-based algorithm

Supplementary Material

Supplementary Table S1: ATC-5 Codes

A. APM medication for inclusion criteria

ATC Group name	ATC code	Generic name	Mechanism of action
**Dopa and dopa derivatives**	N04BA01	Duodopa pump/levopar/ Dopicar	
	N04BA02	Levodopa, Benserazide	Dopamine precursor + inhibitor of dopa Decarboxylase
		Levodopa, Carbidopa	
	N04BA03	Levodopa, Carbidopa, Entacapone, Stavelo	Dopamine precursor + peripheral dopa decarboxylase inhibitor + COMT inhibitor
	N04BA07	Pro-duodopa sub cutaneous pump *	
**Amantadine derivatives**	N04BB01	Amantadine	Dopaminergic, anti-viral
**Dopamine agonists**	N04BC01	Bromocriptine	Dopamine agonist
	N04BC02	Pergolide	
	N04BC04	Ropinirole	
	N04BC05	Pramipexole	
	N04BC06	Cabergoline/ Cabaser/Cabotrim	
	N04BC07	Apomorphine	
	N04BC09	Rotigotine *	
**Monoamine oxidase B inhibitors**	N04BD01	Selegiline	MAO B inhibitor
	N04BD02	Rasagiline	
	N04BD03	Xadago /Safinamide	
**Other dopaminergic agents**	N04BX01	Tolcapone *	COMT inhibitor
	N04BX02	Entacapone	
	N04BX04	Opicapone *	

(*) Not found Clalit’s database

B. Anticholinergic medication

ATC code	Generic name	Medication Name
**N04AA02**	Biperiden	Dekinet/Akineton/Biperdien
**N04AA01**	Trihexphenidyl	Rodenal/Partane/Artane
**N04AA03**	Metixene	Tremaril/Tremoquil/Tremarit

**N04AA04**	Procyclidine	Kemadrin/Procyclidine

C. Anti-psychotic medication

**ATC code**	**Generic name**
**N05AL05**	Amisulpride
**N05AX12**	Aripiprazole
**N05AH05**	Asenapine
**N05AX16**	Brexpiprazole
**N05AX15**	Cariprazine
**N05AA01**	Chlorpromazine
**N05AF03**	Chlorprothixen
**N05AH06**	Clotiapine
**N05AH02**	Clozapine
**N05AF01**	Flupentixol
**N05AB02**	Fluphenazine
**N05AD01**	Haloperidol
**N05AX14**	Iloperidone
**N05AA02**	Levomepromazine
**N05AH03**	Olanzapine
**N05AX13**	Paliperidone
**N05AG03**	Penfluridol
**N05AC01**	Periciazine
**N05AB03**	Perphenazine
**N05AG02**	Pimozide
**N05AH04**	Quetiapine (50 mg and above) *
**N05AX08**	Risperidone
**N05AE03**	Sertindole
**N05AL01**	Sulpiride
**N05AB08**	Thioproperazine
**N05AC02**	Thioridazine
**N05AL03**	Tiapride
**N05AB06**	Trifluoperazine
**N05AE04**	Ziprasidone
**N05AF05**	Zuclopenthixol
**N05AE05**	Lurasidone *
**N05AG01**	Fluspirilene *

(*) Not found in Clalit's database

Supplementary Table S2. The algorithmic selection process results.

	Males		Females		Total
No. of individuals based on the inclusion criteria	27,557		46,397		
	**Number of individuals excluded during the filtering process**				
**Exclusion criterion**	**No.**	**Mean age at index**	**No.**	**Mean age at index**	**No.**
(1) Diagnosed with hyperprolactinemia or pituitary adenoma and never been diagnosed with PD	1374	50.4	3410	35.2	4784
(2) Diagnosed with pituitary adenoma and PD, and exclusively purchased bromocriptine or pergolide.	–	–	–	–	–
(3) Subjects with pituitary adenoma (with PD diagnosis) and index date is in the last ten years (index date>= 2014) that purchased Cabergoline prescriptions exclusively	8	63.8	11	47.6	19
(4) Female patients who exclusively purchased dopamine agonists, never diagnosed with PD, and were either pregnant or gave birth within two years prior to the FPD	NA	NA	20954	29.9	20954
(5) Diagnosed with Hydrocephalus or Anoxic brain diagnosis before and up to one month after the FPD	610	70.3	318	71.6	928

(6) Diagnosed with traumatic brain injury and coma diagnoses within 6 months before the FPD.	2	66.9	2	47.8	4
(7) Diagnosed with one of the following diagnoses: supranuclear palsy (PSP), multiple system atrophy (MSA), or corticobasal degeneration (CBD) with no subsequent PD diagnosis after the last such diagnosis.	45	68.7	38	67	83
(8) Diagnosed with RLS diagnosis with no subsequent PD diagnosis after the last such diagnosis	448	66.7	601	68.5	1049
(9) Purchased at least one anti-psychotic prescription during the year prior to the FPD. (except for Quetiapine under 50 mg) and meet either of the following criteria:<br>• **N**ever purchased Dopa and dopa derivatives.<br>• Purchased Dopa and dopa derivatives and never been diagnosed with PD	1435	61.7	1690	72.4	3125
(10) Exclusively purchased monoamine oxidase (MAO) inhibitors and meet either of the following criteria:<br>• More than 20 purchases or a treatment duration exceeding 5 years;<br>• More than 8 purchases or a treatment duration exceeding 2 years; And never been diagnosed with PD	313	73.9	249	74.4	562
(11) Exclusively purchased Amantadine and meet either of the following criteria:	650	67.6	612	72.2	1262

• More than 20 purchases or a treatment duration exceeding 5 years;<br>• More than 8 purchases or a treatment duration exceeding 2 years;<br>And never been diagnosed with PD.					
(12) Never been diagnosed with PD, purchased APM for more than 2 years and received any of the following diagnoses**:** recurrent falls**,** lethargy**,** or general deterioration**,** within a window of 1 year before to 5 years after the FPD.	249	78.3	266	78.9	515
(13) Never been diagnosed with PD, purchased APM for less than 2 years from the FPD, with more than 1 year having passed since the last APM purchase	1433	67	1,754	68.3	3187
(14) Purchased APM for less than 6 months, with more than 3 years having passed since the last purchase (counted up to the current date, date of death, or HMO exit date).	701	75.8	707	76.8	1408
After the index date change, their index date was before 1.1.2005	857	74.7	860	75.6	1717
**Total PD patients**	19435	74.96	14933	75.48	34368

Abbreviations: APM: antiparkinsonian medication; FPD- first purchase date.

Supplementary Table S3. Possible criteria distribution: The number of PD patients that met each specific criterion.

**Criterion for Possible PD**	**Male**	**Female**	**Total**	**Total sole criterion**
Never received a PD diagnosis	1,913	1,930	3,843	1,475
Less than 2 years have passed from FPD to death date/left the HMO/data extraction date	4,110	2,755	6,865	4,494
Purchased APM over 3 years, and more than 2 years have passed since their last APM purchase	937	1,246	2,183	1,485
Diagnosed with PD and purchased at least one purchase of anti-psychotic prescription during the year before FPD and at least one purchase of Dopa and dopa derivatives medications.	1,167	1,449	2,616	1,915
Total number meeting 3 or 4 possible criteria	-	-	-	-
Total number meeting only 2 possible criteria	1,531	1,538	3,069	-
Total number meeting only 1 possible criterion	5,065	4,304	9369	-
Total Possible	6,5,96	5,842	12,438	

Abbreviations: APM: antiparkinsonian medication; FDP- first purchase date.

Supplementary Table S4. Detailed Classification outcomes

A. PD patients' validation sets

		Identified as PD			Not identified as PD						
Database	No.	Probable PD	Possible PD	Total PD identified	APM<2[a]	#5[b]	#8[b]	#10[b]	#13[b]	#14[b]	Total
Dopa PET Scan	172	150	13	163	4	1	1	1		2	9
Private clinic	383	342	21	363	15	1		2	1	1	20
Movement Disorder outpatient clinic	225	208	5	213	11	1					12

a. Purchased fewer than 2 APM prescriptions (none or one)

b. Columns labeled with #5[b], #8[b], etc. correspond to the specific exclusion steps in the algorithm

B. Mimic cohort

		Identified as PD			Not identified as PD			
Database	No.	Probable PD	Possible PD	Total	APM<1	APM=1	Other exclusion criteria	Total
Mimic cohort-private clinic	128	13	6	19	4	1	1	109

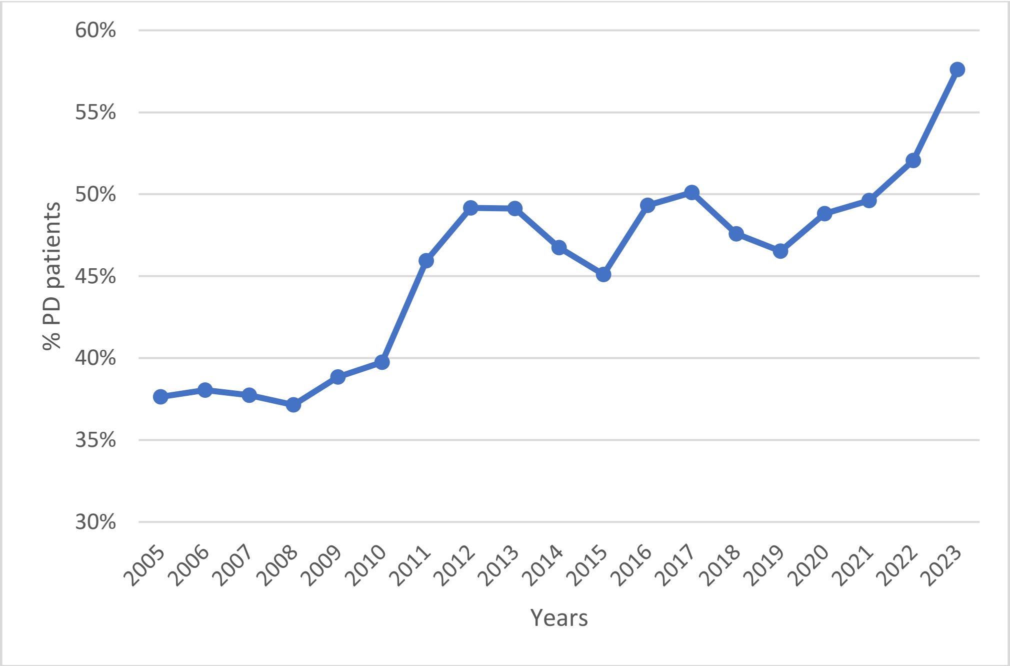

Supplementary Fig. S1: Proportion of PD patients who were diagnosed by a neurologist within the first year after the index date.

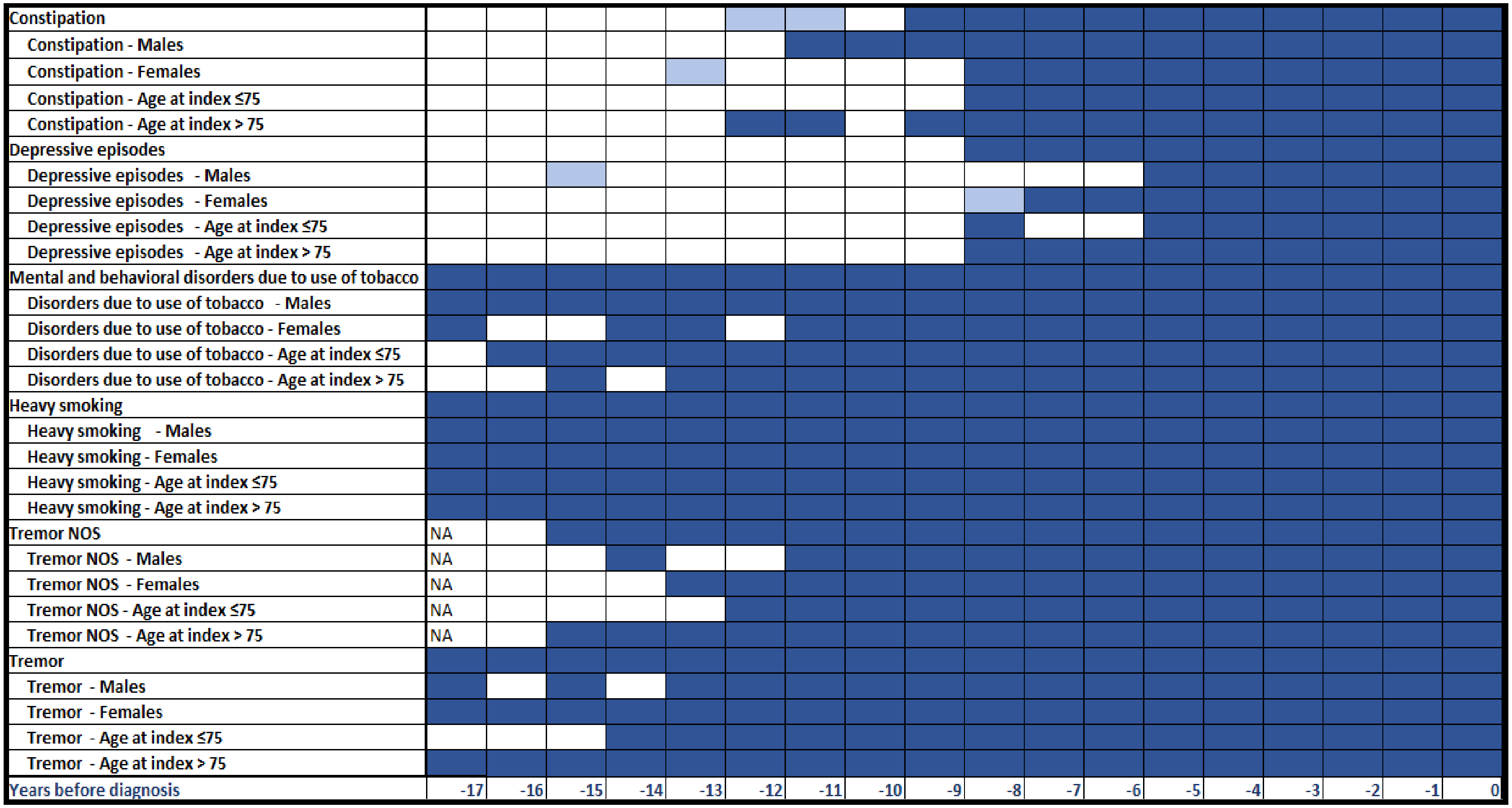

Supplementary Fig. S2: Statistical analysis results of the comparison between the prevalence of diagnoses between PD patients and controls for each year before the index date, up to 18 years (-17) before index date. For all the population and segmentized by sex (Male/Female) and age at index (age at index ≤ 75 and age at index > 75). Colors denote significance levels: FDR-adjusted $p < 0.05$ (dark blue), $p < 0.05$ but FDR-adjusted $p \geq 0.05$ (light blue), and not significant (white).

Supplementary Table S5. p-values, FDR-adjusted p-values, and 95% CIs for the annual comparison of different motor and non- motor diagnosis in the years prior to the index date.

	Constipation			Tremor			Tremor NOS		
**Year before diagnosis**	**p-value**	**Adjusted p-value**	**CI**	**p-value**	**Adjusted p-value**	**CI**	**p-value**	**Adjusted p-value**	**CI**
-17	0.289	0.347	[0.00,0.01]	<0.001 *	<0.001 *	[0.00,0.01]			
-16	0.364	0.409	[-0.01,0.00]	0.001 *	0.001 *	[0.00,0.00]	0.628	0.628	[0.00,0.00]
-15	0.401	0.420	[-0.01,0.00]	<0.001 *	<0.001 *	[0.00,0.00]	0.012	0.014	[0.00,0.00]
-14	0.041 *	0.056	[0.00,0.01]	<0.001 *	<0.001 *	[0.00,0.00]	0.020 *	0.021 *	[0.00,0.00]
-13	0.420	0.420	[0.00,0.01]	<0.001 *	<0.001 *	[0.00,0.00]	0.008 *	0.009 *	[0.00,0.00]
-12	0.027 *	0.043 *	[0.00,0.01]	<0.001 *	<0.001 *	[0.00,0.00]	<0.001 *	<0.001 *	[0.00,0.00]
-11	0.040 *	0.056	[0.00,0.01]	<0.001 *	<0.001 *	[0.00,0.01]	<0.001 *	<0.001 *	[0.00,0.01]
-10	0.065	0.083	[0.00,0.01]	<0.001 *	<0.001 *	[0.00,0.01]	<0.001 *	<0.001 *	[0.00,0.01]
-9	<0.001 *	0.001*	[0.00,0.01]	<0.001 *	<0.001 *	[0.00,0.01]	<0.001 *	<0.001 *	[0.00,0.01]
-8	<0.001 *	<0.001 *	[0.01,0.01]	<0.001 *	<0.001 *	[0.01,0.01]	<0.001 *	<0.001 *	[0.00,0.01]
-7	<0.001 *	<0.001 *	[0.01,0.01]	<0.001 *	<0.001 *	[0.01,0.01]	<0.001 *	<0.001 *	[0.01,0.01]
-6	<0.001 *	<0.001 *	[0.01,0.01]	<0.001 *	<0.001 *	[0.01,0.01]	<0.001 *	<0.001 *	[0.01,0.01]
-5	<0.001 *	<0.001 *	[0.01,0.02]	<0.001 *	<0.001 *	[0.01,0.01]	<0.001 *	<0.001 *	[0.01,0.01]
-4	<0.001 *	<0.001 *	[0.01,0.02]	<0.001 *	<0.001 *	[0.01,0.02]	<0.001 *	<0.001 *	[0.01,0.02]
-3	<0.001 *	<0.001 *	[0.01,0.02]	<0.001 *	<0.001 *	[0.01,0.02]	<0.001 *	<0.001 *	[0.02,0.02]
-2	<0.001 *	<0.001 *	[0.02,0.02]	<0.001 *	<0.001 *	[0.02,0.02]	<0.001 *	<0.001 *	[0.03,0.03]
-1	<0.001 *	<0.001 *	[0.02,0.03]	<0.001 *	<0.001 *	[0.03,0.03]	<0.001 *	<0.001 *	[0.05,0.05]
0	<0.001 *	<0.001 *	[0.04,0.04]	<0.001 *	<0.001 *	[0.06,0.07]	<0.001 *	<0.001 *	[0.19,0.19]

	Depressive episode			Mental and behavioral disorders due to use of tobacco			Heavy smoker		
**Year before diagnosis**	**p-value**	**Adjusted p-value**	**CI**	**p-value**	**Adjusted p-value**	**CI**	**p-value**	**Adjusted p-value**	**CI**
-17	0.597	0.716	[0.00,0.01]	0.045 *	0.045 *	[0.00,0.00]	<0.001 *	<0.001 *	[-0.03,-0.01]
-16	0.640	0.720	[0.00,0.01]	0.011 *	0.012 *	[0.00,0.00]	<0.001 *	<0.001 *	[-0.03,-0.02]
-15	0.228	0.411	[-0.01,0.00]	<0.001 *	<0.001 *	[-0.01,0.00]	<0.001 *	<0.001 *	[-0.04,-0.03]
-14	0.863	0.863	[0.00,0.00]	<0.001 *	<0.001 *	[-0.01,0.00]	<0.001 *	<0.001 *	[-0.03,-0.02]
-13	0.555	0.713	[0.00,0.00]	<0.001 *	<0.001 *	[-0.01,0.00]	<0.001 *	<0.001 *	[-0.03,-0.02]
-12	0.298	0.458	[0.00,0.01]	<0.001 *	<0.001 *	[0.00,0.00]	<0.001 *	<0.001 *	[-0.03,-0.02]
-11	0.791	0.838	[0.00,0.00]	<0.001 *	<0.001 *	[0.00,0.00]	<0.001 *	<0.001 *	[-0.02,-0.02]
-10	0.305	0.458	[0.00,0.00]	<0.001 *	<0.001 *	[-0.01,0.00]	<0.001 *	<0.001 *	[-0.03,-0.02]
-9	0.397	0.549	[0.00,0.00]	<0.001 *	<0.001 *	[0.00,0.00]	<0.001 *	<0.001 *	[-0.02,-0.02]
-8	<0.001 *	0.001 *	[0.00,0.01]	<0.001 *	<0.001 *	[0.00,0.00]	<0.001 *	<0.001 *	[-0.02,-0.02]
-7	0.001 *	0.003*	[0.00,0.01]	<0.001 *	<0.001 *	[-0.01,0.00]	<0.001 *	<0.001 *	[-0.02,-0.02]
-6	0.001*	0.001 *	[0.00,0.01]	<0.001 *	<0.001 *	[-0.01,0.00]	<0.001 *	<0.001 *	[-0.02,-0.02]
-5	<0.001 *	<0.001 *	[0.01,0.01]	<0.001 *	<0.001 *	[0.00,0.00]	<0.001 *	<0.001 *	[-0.02,-0.02]
-4	<0.001 *	<0.001 *	[0.01,0.01]	<0.001 *	<0.001 *	[-0.01,0.00]	<0.001 *	<0.001 *	[-0.02,-0.01]
-3	<0.001 *	<0.001 *	[0.01,0.02]	<0.001 *	<0.001 *	[-0.01,-0.01]	<0.001 *	<0.001 *	[-0.02,-0.01]
-2	<0.001 *	<0.001 *	[0.02,0.03]	<0.001 *	<0.001 *	[-0.01,-0.01]	<0.001 *	<0.001 *	[-0.02,-0.02]
-1	<0.001 *	<0.001 *	[0.03,0.04]	<0.001 *	<0.001 *	[-0.01,-0.01]	<0.001 *	<0.001 *	[-0.02,-0.02]
0	<0.001 *	<0.001 *	[0.08,0.09]	<0.001 *	<0.001 *	[-0.01,-0.01]	<0.001 *	<0.001 *	[-0.02,-0.01]

p-values and FDR-adjusted p-values < 0.05 are marked with an asterisk.

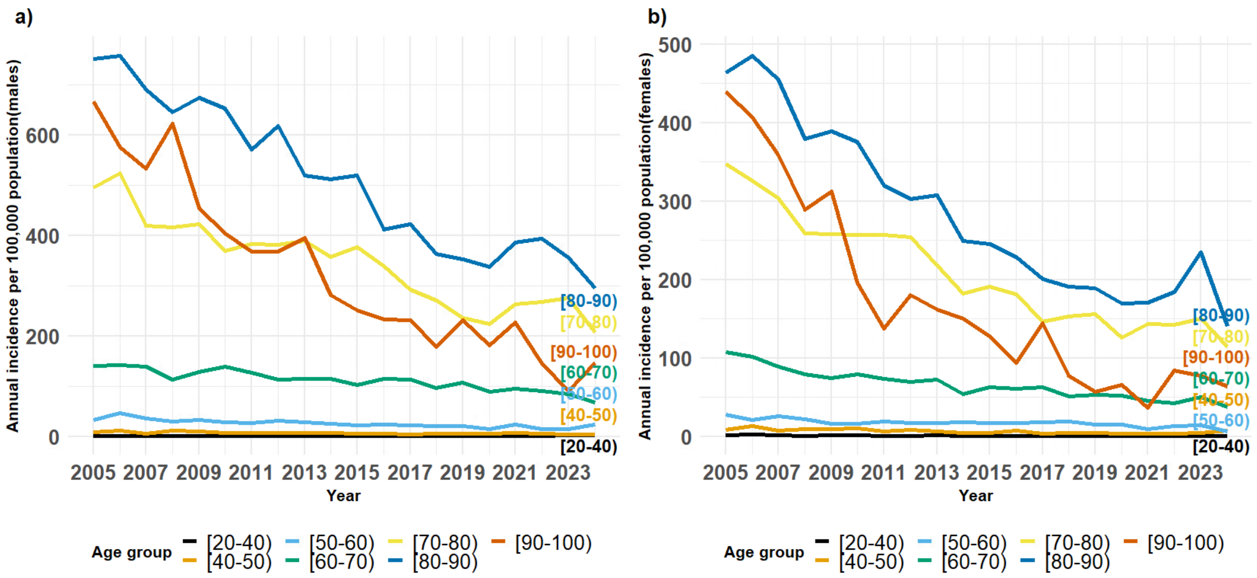

Supplementary Fig. S3. Annual incidence rate of PD per 100,000 CHS insured individuals from 2005 to 2024, stratified by age group **(A)** Males (B) Females

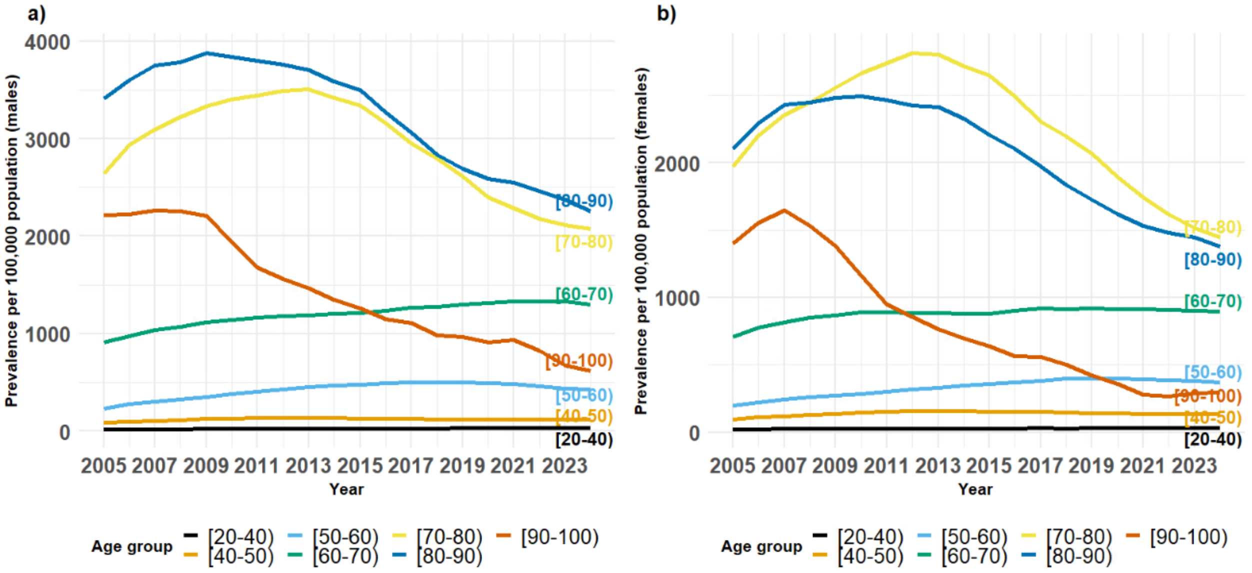

Supplementary Fig. S4. Annual prevenance for PD for CHS insured individuals from 2005 to 2024. **(A)** Males **(B)** Females.
